# Intranasal Premedication Effect of Dexmedetomidine Versus Midazolam on the Behavior of 2-6-Year-Old Uncooperative Children in Dental Clinic

**Published:** 2018-03

**Authors:** Alireza Mahdavi, Masoud Fallahinejad Ghajari, Ghassem Ansari, Leila Shafiei

**Affiliations:** 1 Assistant Professor, Department of Anesthesia, School of Medicine, Shahid Beheshti University of Medical Sciences, Tehran, Iran; 2 Professor, Dental Research Center, Research Institute of Dental Sciences, School of Dentistry, Shahid Beheshti University of Medical Sciences, Tehran, Iran; 3 Assistant Professor, Department of Pediatric Dentistry, Kerman University of Medical Sciences, Kerman, Iran

**Keywords:** Premedication, Intranasal Administration, Midazolam, Dexmedetomidine, Sedation, Dental Care for Children

## Abstract

**Objectives:**

The aim of this study was to compare the intranasal premedication effect of newly introduced dexmedetomidine (DEX) versus midazolam on the behavior of uncooperative children in the dental clinic.

**Materials and Methods:**

This crossover double-blind clinical trial was conducted on 20 uncooperative children aged 2–6 years who required at least two similar dental treatment visits. The subjects were randomly given 1 μg/kg of DEX and 0.5 mg/kg of midazolam via the intranasal route. For the sedation protocol in the two groups, 0.25 mg/kg of atropine in combination with 0.5 mg/kg of midazolam added to 1–2 mg/kg of ketamine were used 30 minutes after premedication and transferring the patient to the operating room. Dental treatments were carried out by a pediatric dentist blinded to the type of the administered premedication. The sedative efficacy (overall success rate) of the agents was assessed by two independent pediatric dentists based on the Houpt scale. Data analyses were carried out according to Wilcoxon signed-rank test and paired t-test.

**Results:**

There were no significant differences in the premedication efficacy of intranasal DEX and midazolam according to the Houpt scale (P>0.05).

**Conclusions:**

Intranasal midazolam and DEX are satisfactory and effective premedication regimens for uncooperative children.

## INTRODUCTION

Sedation is one of the most common methods practiced in dentistry for the treatment of children and adults who are anxious or fearful towards dental procedures to make the process as painless as possible [1]. One of the tasks for pediatric anesthesiologists is to reduce the child’s distress in the operating room and to ease the induction of anesthesia. This is often accomplished by administration of a sedative agent before transferring the child to the operating room. Various pharmacological agents have been used through different methods for sedation in pediatric dentistry. Every method of administration has some advantages and disadvantages [2]. The nasal administration of certain medications has been proven earlier to be highly effective. The nasal administration will allow a high level of absorption because of the high frequency of blood vessels in the region [3–5]. This method will enable the operator to overcome the hardly accepted intravenous (IV) injection as well as the first-pass hepatic metabolism which is believed to reduce the sedative effect of the orally administered medications [3–5]. The blood-brain barrier (BBB) reduces the penetration of medications into the brain through several known pathways [2].

Intranasal drug delivery bypasses the classic BBB obstacles by penetrating into the cribriform plate and by exploiting the paracellular and active neuronal pathways [2]. For many years, midazolam has been administered as one of the safest yet effective medications for sedating children before dental procedures, diagnostic procedures, and medical operations. There has been evidence supporting the superiority of midazolam as an effective tool over the parents’ presence in the operatory or the use of a placebo prior to the induction of general anesthesia [4–9]. In addition to sedation, midazolam has the potential of reducing anxiety during the operation plus lowering the chances of vomiting and nausea after the procedure ends [4–9]. During the recent years, dexmedetomidine (DEX), a newly developed drug, has been used clinically for sedation in dentistry. It has been used in the field of medicine since 1999, mainly for sedation during intubation, in intensive care unit (ICU) patients, and as a premedication.

However, this drug was introduced to dentistry after 2005 [9–14]. DEX is a central α-2 agonist similar to clonidine; however, it is eight times more specific for the central α-2 receptor. This agent is said to induce a milder respiratory depressant effect compared to other available sedative drugs, especially in children [14]. The most distinguishing characteristic of DEX is the high quality of its hypnotic action. Specifically, unlike other available sedatives, it has been described to induce a state similar to physiological sleep [14]. In dentistry, DEX is mainly applied for sedation during third molar surgery and implant surgery. It is also used for sedating children during treatment under general anesthesia [14]. There are several articles on the use of DEX for sedation in dental patients. A recent study reported that intranasal DEX was comparable to intranasal midazolam in producing pre-procedural sedation in children undergoing general anesthesia for complete dental rehabilitation [14]. However, few studies have evaluated the efficacy of DEX in dental procedures, especially as a premedication regimen in children [13,14]. The present study aimed to compare the intranasal premedication effect of DEX versus midazolam on the behavior of 2–6-year-old uncooperative children in the dental clinic.

## MATERIALS AND METHODS

**Fig. 1:**
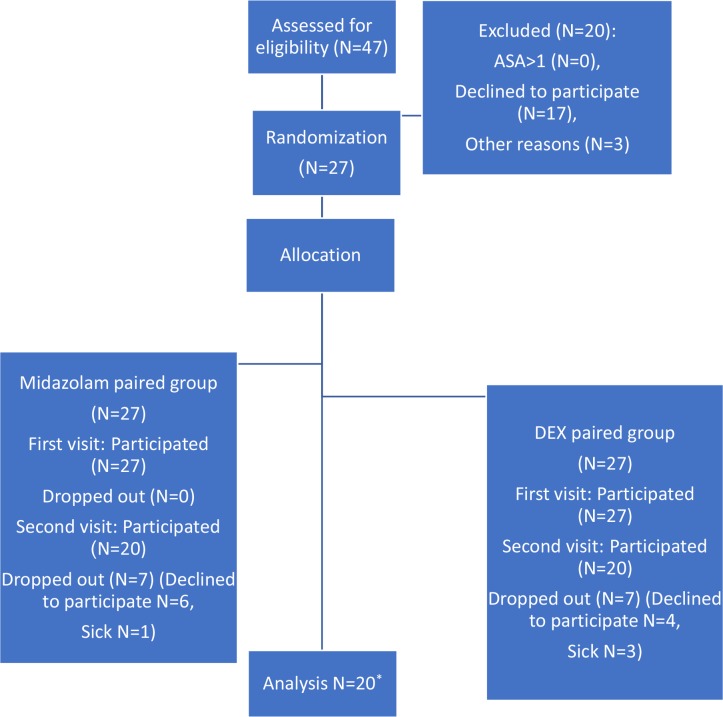
Consort diagram: the different phases of the trial from enrolment to data analysis in the two groups. *Twenty subjects were analyzed according to a paired design (20 readings for each of the two groups, a total of 40 readings). DEX=Dexmedetomidine

This randomized crossover double-blind clinical trial (IRCT No: 201601281882N7) was conducted on 20 children aged 2–6 years who were referred to the department of pediatric dentistry of school of dentistry of Shahid Beheshti University of Medical Sciences. Uncooperative 2–6-year-old children with negative or definitely negative behaviors according to the Frankl behavioral rating scale and with health classification of ASA I in the American Society of Anesthesiologists (ASA) physical status classification system were included in this study for dental treatments under deep sedation [15]. We used the Minitab software (Minitab Inc., Pennsylvania, USA) with the mean sedation level=9.5%, alpha=0.05, and beta=0.2 for sample size determination. The sample size calculations showed that we needed 16 patients for the study. By considering a potential loss of samples equal to 20%, we targeted to recruit 20 participants for the study [7] (Fig. 1). All the patients were booked for two separate sessions of pulpotomy and restoration with stainless steel crown (SSC) or other restorative materials. The subjects had no signs of systemic problems, common cold, or nasal or respiratory infections. Also, there were no signs of enlarged tonsils or problems in neck movements or tongue size. A written informed consent was received from the patients’ parents or legal guardians accompanied by comprehensive pre-sedation instructions. The stages of sedation were performed under the direct supervision of an anesthesiologist.

The primary vital signs of oxygen saturation (SpO_2_), respiratory rate (RR), heart rate (HR), and blood pressure (BP) were recorded at the start and at the end of the treatment session. All the subjects were kept at NPO (nothing by mouth) status for 6 hours (solid foods) and 4 hours (water and liquids) before the operation. The children were randomly assigned to two groups of A: intranasal sedation with midazolam, and B: intranasal sedation with DEX. A questionnaire was used to record the medical and dental histories. In group A, 0.5 mg/kg of midazolam (Chemidaru Industrial Co., Tehran, Iran) was dropped into the nostrils, while in group B, 0.1 μg/kg of DEX (Precedex®, Hospira, Lake Forest, IL, USA) was dropped into the nostrils by a blinded pediatric dentist by using an insulin syringe [15,16]. For the sedation protocol in the two groups, 0.25 mg/kg of atropine (Abu Reyhan Pharmaceutics Co., Tehran, Iran) in combination with 0.5 mg/kg of midazolam (Chemidaru Industrial Co., Tehran, Iran) plus 1–2 mg/kg of ketamine (Chemidaru Industrial Co., Tehran, Iran) were used 30 minutes after premedication and transferring the patient to the operating room. Both groups received the alternate treatment regimen during their second session which was one week after the first session. This was aimed to have every child serve as his/her own control. Local anesthesia was achieved by administrating a cartridge of 2% lidocaine hydrochloride with 1:80000 epinephrine (Darou Pakhsh Pharmaceutical Mfg. Co., Tehran, Iran). Dental treatments were carried out by a pediatric dentist blinded to the type of the administered premedication. The sedative effect of the drugs was measured after 30 minutes. A standard flow of O_2_ was administered to all the patients throughout the procedure. The SpO_2_ was monitored at various stages of the study, starting with the premedication time (the baseline), by using a multipurpose monitoring unit (Saadat Co, Tehran, Iran). All the measurements were made at the baseline, at the IV puncture time, and at the end of the dental treatment. The Houpt scale was used to record the changes in the children’s behavior according to the following categories: the amount of crying (C), sleeping (S), movement (M), and overall behavior (O) [15]. Video recordings were also performed during the entire treatment session, which were later evaluated and scored by a pediatric dentist blinded to the administration of drugs. The total amount of ketamine used for each patient was recorded. Attempts were made to limit each treatment session to a maximum of 35 minutes. The children were discharged when full consciousness was regained (judged by the anesthesiologist) and all the vital signs returned to the normal range. The parents were interviewed 24 hours after each session with regard to a series of questions in relation to postoperative complications. Wilcoxon signed-rank test was used for statistical analyses of the data related to the Houpt scale, while paired t-test was used for analyzing the data related to the vital signs at a significance level of 0.05.

## RESULTS

Data related to the twenty 2–6-year-old children (12 boys and 8 girls) were recorded. The mean age of the patients was 4.1±11.14 years, and the mean weight was 14.51±3.09 kg. The initial evaluation of the Frankl behavioral rating scale revealed that 14 cases (70%) were definitely negative, while 6 cases (30%) were judged to be negative. Overall, no significant differences were found between the administered drugs (P=0.250). The comparison of sleep (S), movement (M), crying (C), and overall behavior (O) parameters showed no significant differences between the two groups at the IV puncture time (P>0.05, Tables 1 to 4). As mentioned earlier, all the participants were selected from among those classified as definitely negative and negative, and the drugs were administered by force in both sessions. Since each patient also served as his/her control, comparison of the outcomes showed no difference in the drug acceptance rate (P=0.225).

**Table 1.** Distribution of patients in terms of their sleepiness in the two groups based on the Houpt Scale

**Sleepiness scores**	**DEX**		**MID**	

	N	%	N	%
**1-Fully awake and alert**	2	10	0	0
**2-Dizzy and sleepy**	11	55	10	50
**3-Sleepy**	7	35	10	50
**Total**	20	100	20	100

DEX=Dexmedetomidine, MID=Midazolam

**Table 2.** Distribution of patients in terms of their movement in the two groups based on the Houpt Scale

**Movement score**	**DEX**		**MID**	

	N	%	N	%
**1-Violent and disruptive to treatment**	1	5	1	5
**2-Continous, making treatment difficult**	6	30	1	5
**3-Controllabale, no interruption**	5	25	6	30
**4-No movement**	8	40	12	60
**Total**	20	100	20	100

DEX=Dexmedetomidine, MID=Midazolam

**Table 3.** Distribution of patients in terms of their cry in the two groups based on the Houpt Scale

**Cry score**	**DEX**		**MID**	

	N	%	N	%
**1-Hysterical**	4	20	1	5
**2-Continous, making treatment difficult**	4	20	2	10
**3-Intermittent and mild**	4	20	5	25
**4-No crying**	8	40	12	60
**Total**	20	100	20	100

DEX=Dexmedetomidine, MID=Midazolam

**Table 4.** Frequency and percentage of overall behavior in the two groups

**Overall behavior**	**DEX**		**MID**	

	**N**	**%**	**N**	**%**
**1-No treatment**	0	0	0	0
**2-Treated partially (stopped)**	3	15	0	0
**3-Treatment completed despite interruption**	4	20	3	15
**4-Difficult but done**	4	20	6	30
**5-Little crying and movement**	8	40	1	5
**6-No crying or movement**	1	5	10	50
**Total**	20	100	20	100

DEX=Dexmedetomidine, MID=Midazolam

According to the parents, the most common postoperative complicatios were nausea, vomiting, drowsiness, and reduced activity during the initial 24 hours after the sessions involving midazolam or DEX premedication with no significant differences. The total amount of ketamine administered through the IV line for deep sedation was higher in the midazolam group compared to the DEX group; however, the difference was not significant (P=0.140). There were no significant statistical differences between the two groups with regard to the HR (P=0.150), SpO_2_ (P=0.534), RR (P=0.337), and maximum and minimum BP (P=0.157 and 0.239, respectively).

## DISCUSSION

Based on the results of the present research, sedation techniques show a promising potential for resolving the children’s interfering behaviors in the dental office. Both intranasal midazolam and DEX regimens could provide certain levels of calmness for the child and dentist during dental procedures. Based on the results of this investigation, it appears that certain children with different degrees of fear and anxiety will take advantages of various sedation levels, while the others would enjoy current behavioral modification techniques [15]. The best premedication route and agent are still unclear. Midazolam, which induces sedation, anxiolysis, and amnesia, is one of the most common premedication agents [10–13]. It has supplementary valuable properties such as anticonvulsant activity, a rapid onset, and a short duration of action [10–13]. Midazolam is considered as the most popular medication in this respect. It provides an anterograde amnesia much stronger than that provided by other benzodiazepines [13]. Higher bioavailability and a quicker onset have been demonstrated for intranasal midazolam administration [10–15]. However, midazolam is not an ideal premedication due to its adverse effects including restlessness, paradoxical reactions, cognitive impairment, postoperative behavioral changes, respiratory depression, and nasal irritation [13,16]. DEX is a highly selective α-2 adrenoceptor agonist which provides sedation, anxiolysis, and analgesic effects without causing respiratory depression, especially in pediatric patients. Therefore, it has been explored extensively in the pediatric population [13]. Oral sedation is the most common and easily accepted technique among the several methods of sedation in children. However, a delayed onset is the main disadvantage of oral sedation. Other disadvantages include a long recovery time and high first-pass metabolism. The highest level of effect is usually reached after 40–60 minutes of drug administration [15–19]. Among the known routes of drug administration, the nasal route is proven to have the potential of a high absorption rate; therefore, a quick response is expected because of the rich nasal vascular network allowing the medication to rapidly reach the target cells. The children’s compliance with nasal administration is better in comparison with oral sedation. [2,5]. Since each patient served as his/her own control, a chance was provided to omit the interfering factors and to allow for maximum similarities between the two sessions with two different premedication regimens. The minimum dosage of 1 μg/kg of DEX was employed in this investigation with acceptable results in the studied age group. Higher potential effects of DEX may be expected from higher doses (1.5 μg/kg), which in turn, may delay the recovery.

The comparable sedative effects of midazolam and DEX according to the Houpt scale were similar to the results of many other studies such as the study by Sheta et al [17]. Post-sedation side effects were not significantly different in the two groups of the present study, similar to the results reported by Taniyama et al [20], Shirakami et al [21], and Sheta et al [17]. Bhat et al [22] stated that DEX causes smaller degrees of postoperative agitation in children between the ages of one and six years old. In the present study, the dosage of the sedative drugs used through the IV line was recorded and compared as well. Although the difference was not significant, the DEX group received lower amounts of sedative agents compared to the midazolam group, similar to the findings of the study by Attri et al [23]. This difference in dosage suggests the higher sedative effect of DEX; a theory which can be proven through further studies. Peng et al [13] suggested that DEX can be considered as an ideal premedication regimen due to the satisfactory results in comparison with midazolam. We found no differences between the DEX and midazolam groups in hemodynamic stability or oxygenation prior to, during, or after dental procedures, similar to the results of the study by Jannu et al [24]. None of the patients required additional interventions during the procedure since the vital signs were within the normal range. However, the airways, tonsillar size and space, and the type and dosage of sedative agents must be examined carefully to reduce the complications. Knowledge of various sedative routes and regimens is crucial for safe and effective procedures.

## CONCLUSION

1. DEX and midazolam showed comparable premedication efficacies.
2. Both premedication regimens were efficient according to the Houpt scale.
3. All the vital signs remained within the normal range during the procedure, and no interventions were needed.
4. The most common side effects during the first 24 hours were vomiting and dizziness for both premedication regimens.
